# Proteomic Remodeling in the Female Heart During Spaceflight and Recovery

**DOI:** 10.3390/ijms27209215

**Published:** 2026-10-17

**Authors:** Aamena Ahmed, Daniel Figueiredo, Rebekah J. Woolsey, David R. Quilici, Stephanie Puukila, Joshua S. Alwood, April E. Ronca, Lane K. Christenson, Candice G. T. Tahimic

**Affiliations:** 1 Department of Biology, University of North Florida, Jacksonville, FL 32224, USA; n00942484@unf.edu (A.A.); n01470499@unf.edu (D.F.); 2 Nevada Proteomics Center, University of Nevada, Reno, NV 89557, USAquilici@unr.edu (D.R.Q.); 3 Blue Marble Space Institute of Science, Moffett Field, CA 94035, USA; 4 Space Biosciences Division, NASA Ames Research Center, Moffett Field, CA 94035, USA; 5 Department of Cell Biology and Physiology, University of Kansas Medical Center, Kansas City, KS 66160, USA; lchristenson@kumc.edu

**Keywords:** spaceflight, microgravity, heart, proteomics, mitochondria, cellular respiration, metabolism

## Abstract

Spaceflight profoundly impacts cardiovascular physiology. To gain insight into the underlying molecular adaptations, we examined the female cardiac proteome during spaceflight and recovery. We hypothesized that spaceflight remodels the cardiac proteome, including mitochondrial bioenergetics and redox homeostasis pathways, during both flight and recovery, altering cardiovascular health risk. Proteomic analysis was performed on hearts from female C57BL/6J mice flown aboard the International Space Station (ISS) for 55 to 60 days as part of the NASA Rodent Research-20 (RR-20) study and a recovery cohort returned to Earth after 42 days, with sampling occurring five days after landing, and corresponding ground controls. Consistent with our hypothesis, spaceflight enriched mitochondrial bioenergetic pathways relative to ground controls, including cellular respiration, the tricarboxylic acid cycle, and oxidative phosphorylation. These changes occurred alongside the remodeling of antioxidant pathways, suggesting altered redox regulation during flight and increased susceptibility to reactive oxygen species (ROS) accumulation in the heart versus controls. Postflight recovery was characterized by distinct metabolic, redox, and structural remodeling signatures, including pathways associated with sarcomere organization, contractile function, and extracellular matrix regulation. These findings reveal cardiac proteomic remodeling during and after spaceflight and provide molecular insights relevant to cardiovascular risk assessment and countermeasure development for exploration missions.

## 1. Introduction

Spaceflight exposes humans to multiple physiological stressors, including microgravity, intermittent hypergravity during launch and landing, ionizing radiation, confinement, circadian disruption, and dietary changes [1,2]. The cardiovascular system is particularly susceptible because it must continuously adapt to altered gravitational and hemodynamic forces. In microgravity, redistribution of body fluids toward the head and thorax (cephalad fluid shift) increases stroke volume and cardiac output [3,4] amidst overall reductions in activity levels. These hemodynamic changes are accompanied by compensatory reductions in plasma volume [5,6] and alterations in fluid and electrolyte homeostasis [7,8], which contribute to cardiovascular deconditioning if no countermeasures are implemented. Manifestations of cardiovascular deconditioning include cardiac rhythm disturbances [9], transient atrial enlargement [10], reductions in left ventricular mass [11,12], increased carotid artery stiffness [13] and orthostatic intolerance upon the return to Earth [7]. In addition, prolonged spaceflight has been associated with venous blood flow stasis, retrograde venous flow, and thrombus formation [4,14].

Animal models have provided important mechanistic insights into how altered gravity affects the cardiovascular system. Rodent hindlimb unloading (HU), a ground-based analog of microgravity [15,16], reproduces a number of cardiovascular changes observed during spaceflight such as decreased left ventricular mass and volume [17], arrhythmias and aberrant intracellular Ca^2+^ signaling [18], and altered regulation of blood pressure and heart rate [19]. Other studies have also revealed altered redox balance in the heart [20] in a sex-dependent manner [21], with some changes becoming apparent only after a prolonged recovery period compared with time-matched controls [21]. Transcriptomic analyses of rat hearts following HU have revealed differential expression of genes involved in pro-inflammatory signaling, energy metabolism, and cardiovascular disease (CVD) progression [21]. Omics-based analyses from spaceflight studies have extended these findings. Female mice flown aboard the space shuttle for 15 days during the STS-131 mission and euthanized approximately three hours after the return to Earth exhibited altered cardiac gene expression associated with redox regulation, cell cycle control, and senescence [22]. Cardiac proteomic analysis of male mice flown aboard the ISS for approximately 38.5 days as part of the NASA Rodent Research-23 (RR-23) study and euthanized within 18 h of return to Earth revealed alterations in proteins associated with cell adhesion and migration, inflammatory signaling, and vascular disease pathways, whereas proteins involved in sarcomere organization and contractile function were largely unaffected [23]. Complementing these findings, cardiac miRNA profiling of female mice from NASA RR-8 demonstrated altered miRNA signatures in animals euthanized either onboard the ISS (~21 days in orbit) or approximately three days after the return to Earth following 42 days of spaceflight. These miRNA changes were involved in the regulation of extracellular matrix (ECM) remodeling, postsynaptic membrane organization, and potassium ion channel signaling [24]. Collectively, these studies indicate that spaceflight induces coordinated molecular remodeling of the heart involving genes related to inflammation, redox regulation, metabolism, ECM organization, and vascular function, pathways that are also hallmarks of CVD progression [25,26]. However, important gaps remain in our understanding of the molecular adaptations of the female heart to spaceflight and the ensuing recovery. Cardiac proteomic data from female mice remain limited, especially from animals euthanized onboard the ISS, where molecular responses can be evaluated without the physiological influences associated with re-exposure to Earth’s gravity. The current study addresses these knowledge gaps by examining cardiac molecular adaptations during spaceflight and recovery using a proteomic approach. We analyzed hearts from female mice flown aboard the ISS as part of NASA RR-20 [27]. One cohort was euthanized after 55–60 days aboard the ISS, enabling assessment of cardiac adaptations occurring during spaceflight, whereas a second cohort was euthanized approximately five days after the return to Earth, after 42 days of spaceflight to characterize recovery responses. Age-matched control animals were housed on Earth in similar-make habitats. All mice received hormone treatment to induce ovulation 52−54 h before euthanasia to address the primary scientific questions of NASA RR-20 related to female fertility. The resulting synchronization of estrous cycles also provides a unique opportunity to investigate proteomic adaptations to spaceflight in the female heart within a consistent endocrine context. We hypothesized that spaceflight remodels the female cardiac proteome, including mitochondrial bioenergetics and redox homeostasis pathways, during both flight and recovery, thereby altering cardiovascular health risk. To characterize these molecular adaptations, we performed gene set enrichment analysis (GSEA) and evaluated potential changes in telomere dynamics and mitochondrial homeostasis during spaceflight and recovery.

## 2. Results

### 2.1. Distinct Cardiac Proteome Signatures During Spaceflight and Recovery

To investigate the impact of spaceflight on the heart, proteomic analysis was performed on left ventricles from four experimental groups representing two study phases: on-orbit and live animal return (LAR). On-orbit groups comprised mice euthanized aboard the ISS after 55–60 days in space and their age- and habitat-matched ground controls (on-orbit HGC), whereas LAR groups comprised mice euthanized approximately five days after returning to Earth following 42 days in space and their corresponding age- and habitat-matched ground controls (LAR HGC) (refer to Section 4 and Supplementary Methods for study design details). For brevity, comparisons between each flight group and its corresponding HGC are referred to as on-orbit flight vs. HGC and LAR flight vs. HGC. Pairwise comparisons between on-orbit and LAR cohorts were not performed because of substantial differences in tissue preservation (details in Section 4).

**Figure 1:**
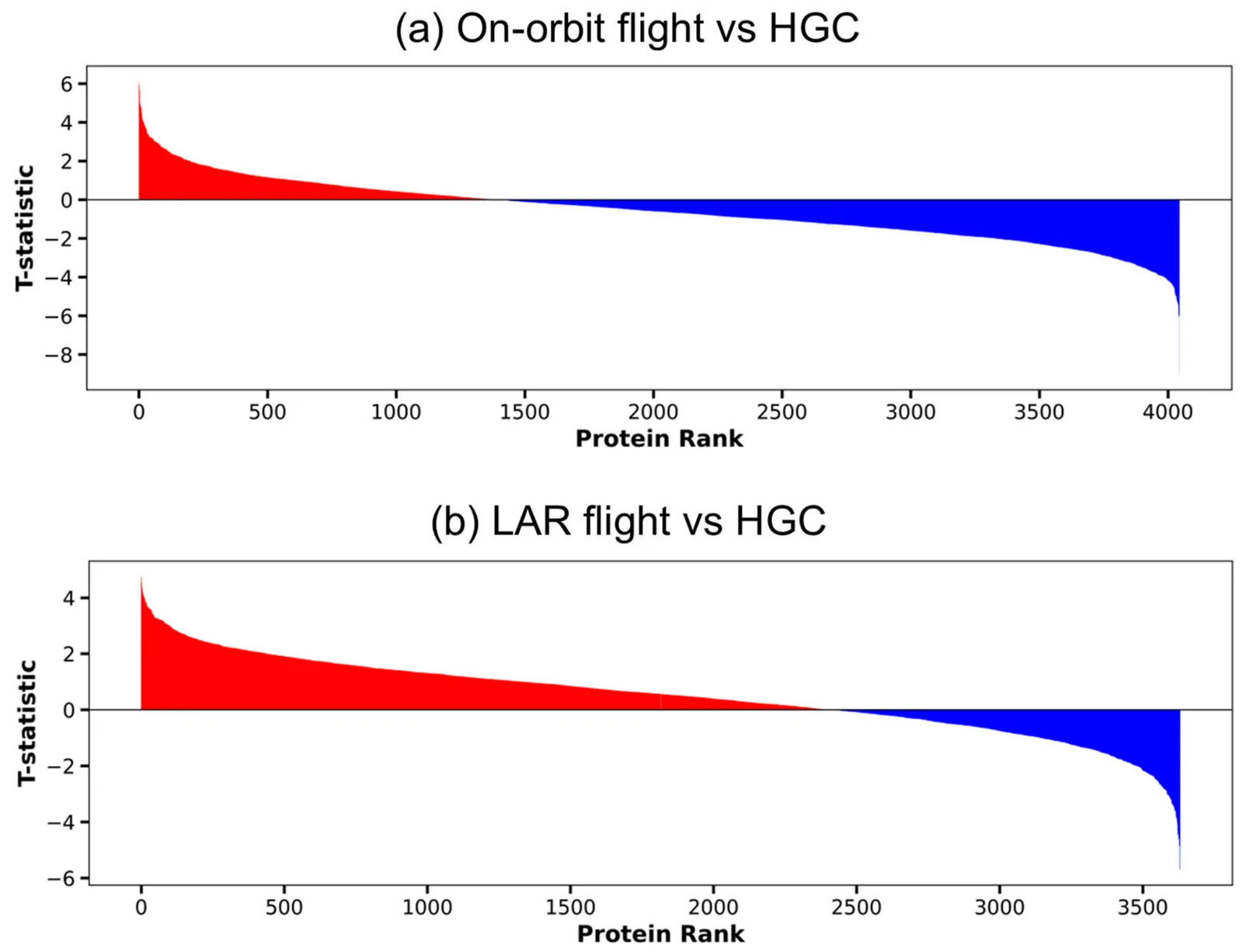
T-statistic distributions of proteins from (**a**) on-orbit flight vs. HGC and (**b**) LAR flight vs. HGC. Red: upregulated, blue: downregulated proteins. On-orbit: N = 7/group, LAR: N = 4/group. Refer to Tables S1 and S2 for the full ranking of proteins.

Following mass spectrometry (MS) analysis and processing, 4046 proteins remained for the on-orbit flight vs. HGC comparison and 3632 for LAR flight vs. HGC. Rank-ordered t-statistic distributions revealed distinct proteomic responses between study phases. The on-orbit flight vs. HGC comparison exhibited a broader range of t-statistics (−9.05 to 6.15; Figure 1a, Table S1) than LAR flight vs. HGC (−5.72 to 4.79; Figure 1b, Table S2). Consistent with these distributions, on-orbit comparisons had more downregulated than upregulated proteins, whereas the opposite pattern was observed for LAR (Figure S1a,b; Tables S1 and S2). Principal component analysis (PCA) showed clustering by treatment in both on-orbit and LAR cohorts (Figure S2a,b).

### 2.2. Cardiac Proteomic Signature During Spaceflight

GSEA revealed 67 significantly enriched gene sets (false discovery rate, FDR < 0.05) for the on-orbit flight vs. HGC comparison, summarized in Table 1. Of these enriched gene sets, the largest functional categories were regulation of gene expression (25 gene sets), energy metabolism and mitochondrial bioenergetics (13 gene sets), non-CVD processes, and other metabolic processes (eight gene sets each).

In this analysis, a positive normalized enrichment score (NES) indicates that leading edge proteins are enriched in the flight group relative to the corresponding HGC group, whereas a negative NES indicates enrichment in the HGC group. Enriched gene sets related to regulation of gene expression include nonsense-mediated decay (NES: 3.0) and translation (NES: 2.1) with multiple Reactome pathways related to the regulation of translation (Table S3). Examination of leading edge proteins revealed that enrichment across many of these translation-related pathways was driven predominantly by ribosomal protein large subunit (RPL) proteins. Because Reactome translation pathways share substantial gene overlaps, particularly among RPLs, the large number of enriched translation-related pathways likely reflects annotation redundancy rather than distinct biological processes.

**Figure 2:**
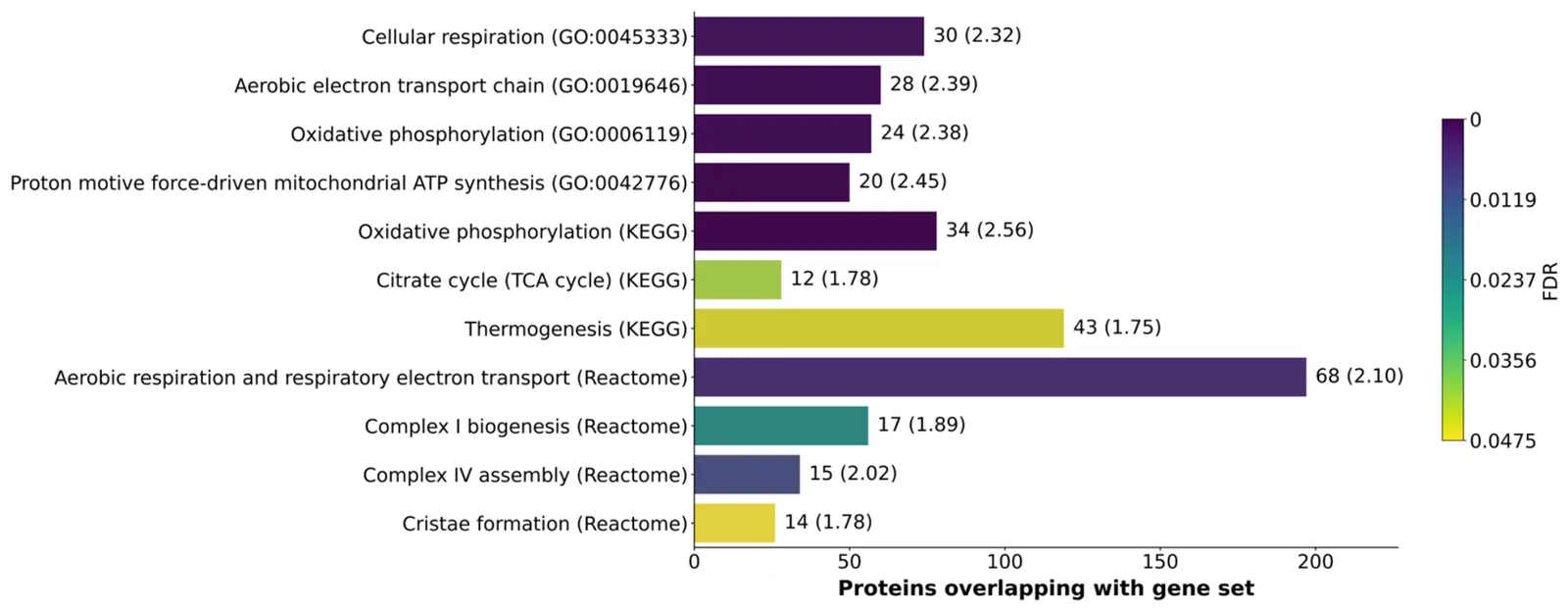
Select significant gene sets involved in energy metabolism and mitochondrial bioenergetics, respiratory chain assembly (Complex I biogenesis and Complex IV assembly), and mitochondrial organization (cristae formation) from GSEA of on-orbit flight vs. HGC (FDR < 0.05). The first value at the end of each bar plot depicts the number of leading edge proteins driving the enrichment signal. The value in parentheses is the NES. Refer to Table S3 for a full list of GSEA gene sets meeting the FDR threshold.

**Figure 3:**
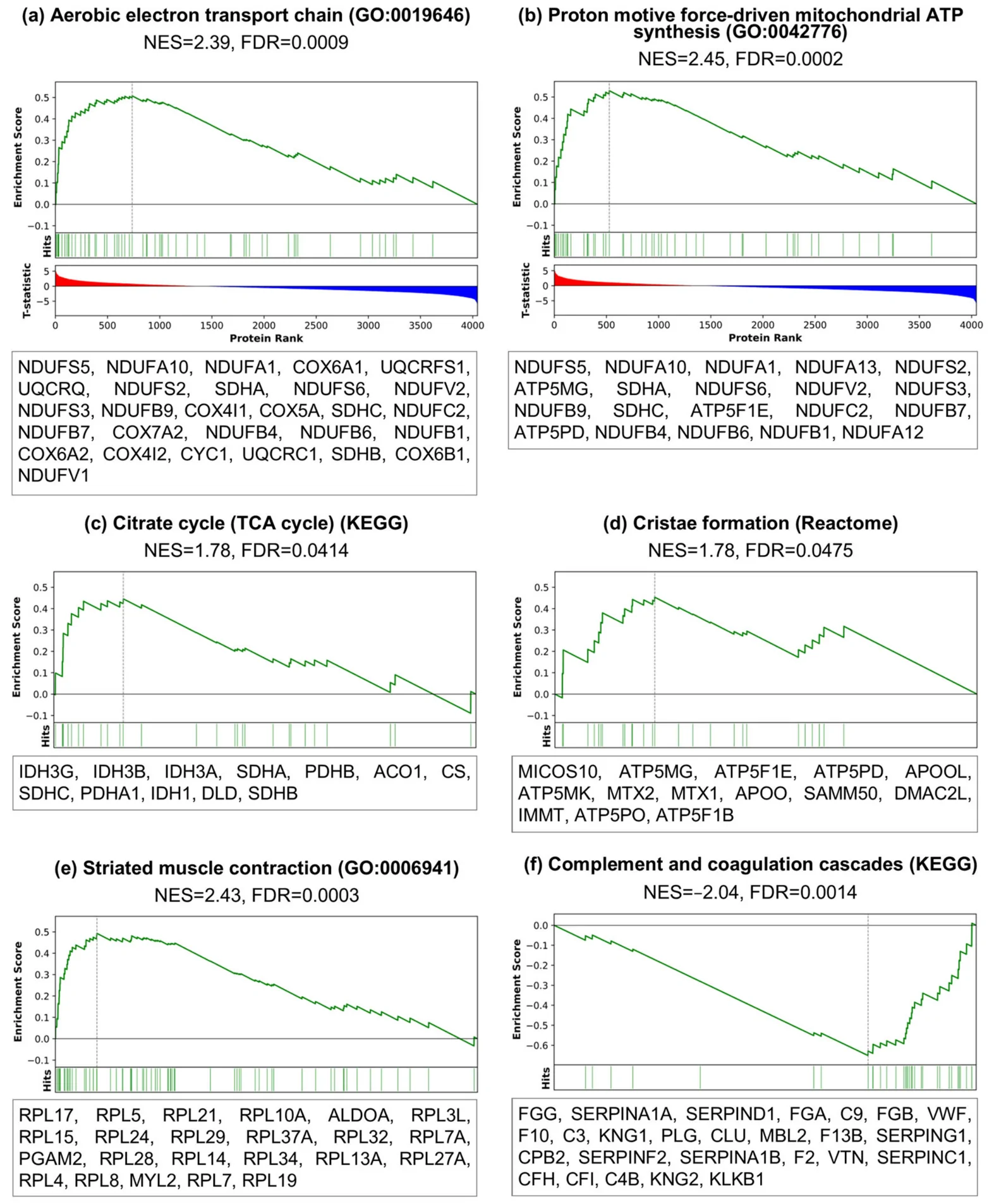
Select GSEA enrichment plots for the on-orbit flight versus HGC comparison showing terms associated with (**a**–**c**) energy metabolism and mitochondrial bioenergetics, (**d**) mitochondrial organization, (**e**) muscle contraction and contractile function and (**f**) immune regulation and response. For each plot, the top panel shows the running enrichment score (RES) of proteins that overlapped with the gene set. The dashed line represents the leading edge protein with the highest RES. Middle panel: Vertical solid green lines denote the position of proteins that overlapped with the gene set plotted along the protein ranking. Protein rankings based on the t-statistic (from Figure 1) are shown in the third visualization panel with ranking applicable to all gene sets but only provided for plots (**a**,**b**) for brevity. Listed are leading edge proteins, from highest to lowest RES.

Gene sets related to energy metabolism and mitochondrial bioenergetics included processes such as cellular respiration, oxidative phosphorylation, and proton motive force-driven mitochondrial ATP synthesis (NES: 2.3 to 2.4) (Figure 2 and Figure 3, Table S3) (reviewed in [28]). Tricarboxylic acid cycle (TCA) and thermogenesis and aerobic electron transport chain (ETC) were also enriched. Gene sets involved in respiratory chain assembly were also enriched specifically, Complex I biogenesis and Complex IV assembly, as well as mitochondrial organization, represented by cristae formation (Figure 2 and Figure 3, Table S3). Notably, leading edge proteins for proton motive force-driven mitochondrial ATP synthesis include NDUF proteins which comprise Complex I, ATP5 proteins which are components of mitochondrial ATP synthase (Complex V) and SDH which comprise succinate dehydrogenase (Complex II), also a key enzyme in the TCA (Figure 3). For the TCA gene set, leading proteins include pyruvate dehydrogenase (PDH) subunits, citrate synthase (CS) which catalyzes the production of citrate from acetyl-CoA and oxaloacetate, and isocitrate dehydrogenase 3 subunits (IDH3) which catalyzes the conversion of isocitrate to α-ketoglutarate, also leading to the production of NADH from NAD^+^ (reviewed in [29]). The cristae formation gene set included leading edge proteins from the MICOS complex, including IMMT, MICOS10, APOO (MIC26), and APOO (MIC27) [30,31]. Additional leading edge proteins included SAMM50, MTX1, and MTX2 [32], which are associated with the sorting and assembly machinery complex involved in mitochondrial β-barrel protein integration into the outer mitochondrial membrane [33].

Gene sets associated with muscle structure and contractile function were also enriched, including regulation of myoblast fusion (NES: 2.9) and striated muscle contraction (NES: 2.4) (Table S3). Leading edge proteins contributing to regulation of myoblast fusion were predominantly RPLs, suggesting that this enrichment may reflect broader changes in protein synthesis-related processes rather than muscle-specific remodeling. Leading edge proteins associated with striated muscle contraction included sarcomeric protein MYL2, as well as glycolytic enzymes ALDOA and PGAM2 (Figure 3e). An immune-related gene set, complement and coagulation cascades, was negatively enriched (NES: −2.0), with leading edge proteins including complement components C3, C4B, and C9, and complement regulatory proteins CFH and CFI (Figure 3f, Table S3).

**Figure 4:**
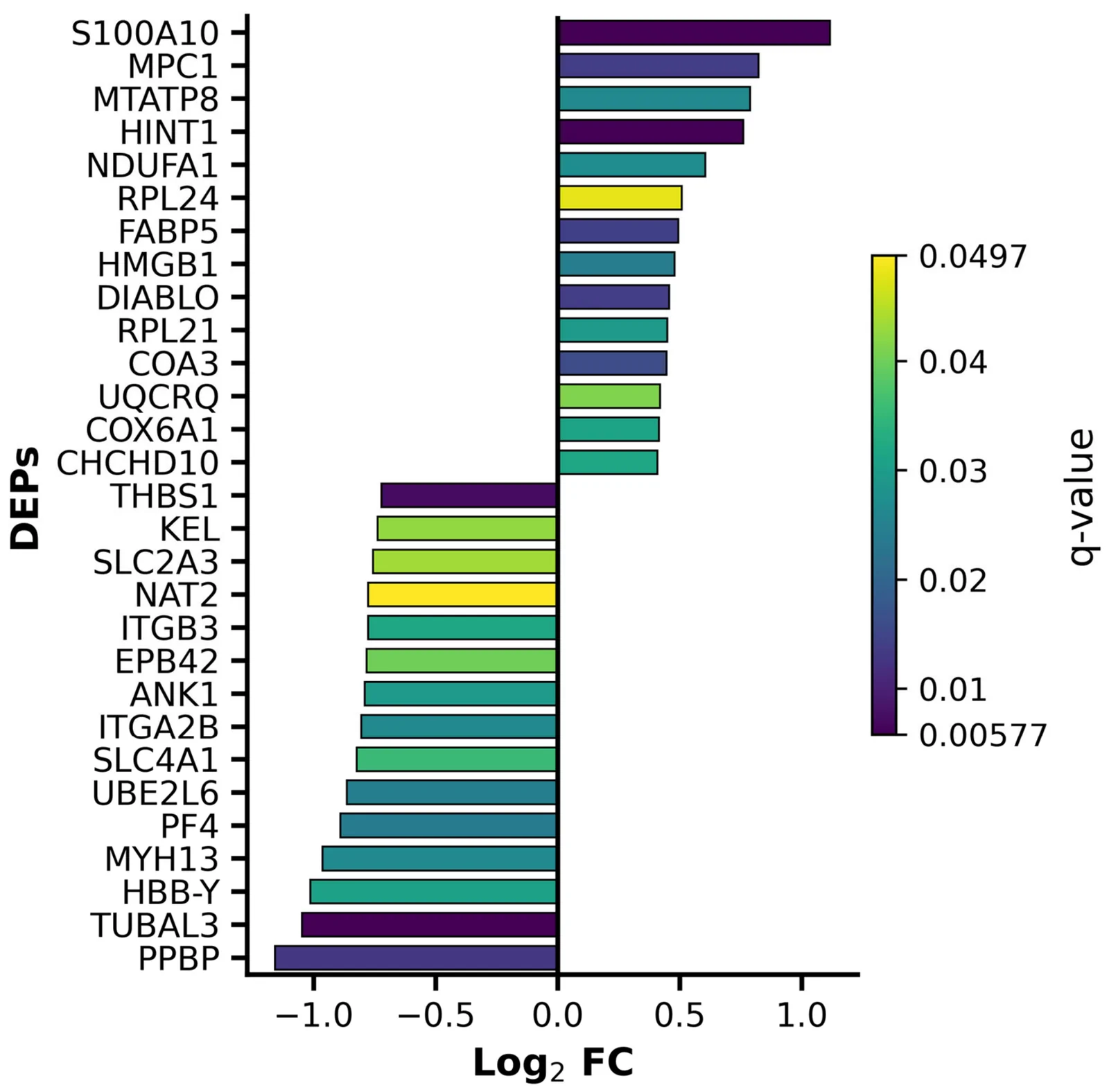
Top DEPs in on-orbit flight relative to HGC. The top 15 downregulated DEPs and all 14 upregulated DEPs are shown. N = 7/group. Refer to Table S1 for a full list of DEPs.

Because GSEA evaluates coordinated changes across the entire ranked proteome, overrepresentation analysis (ORA) was also performed on differentially expressed proteins (DEPs) at |log_2_ fold change (FC)| > 0.4, q-value < 0.05 to identify processes overrepresented among the most significantly altered proteins. In the on-orbit flight relative to the HGC group, there were 101 DEPs (14 upregulated and 87 downregulated) (Table S1). Upregulated DEPs (Figure 4) are predominantly associated with mitochondrial energy metabolism, including components of the ETC (COX6A1, UQCRQ, NDUFA1, MTATP8), Complex IV assembly factor COA3, and mitochondrial pyruvate carrier MPC1. Also upregulated are CHCHD10, involved in mitochondrial cristae organization and integrity [34], DIABLO (aka SMAC) which functions in mitochondrial-mediated apoptosis [35] and a fatty acid-binding protein (FABP5), a chaperone that transports long-chain fatty acids.

Some of the top 15 downregulated DEPs (Figure 4, Table S1) are involved in platelet activation and coagulation such as PF4 (aka CXCL4), PPBP (aka CXCL7), integrins (ITGA2B, ITGB3) and thrombospondin THBS1. Also in the top 15 downregulated genes are HBB-Y, SLC4A1, ANK1, EPB42, and KEL, involved in erythrocyte structure and oxygen binding. The glucose transporter SLC2A3 (aka GLUT3) was also downregulated. Notably, other downregulated DEPs are involved in antioxidant signaling such as catalase (CAT), glutathione peroxidase 1 (GPX1), glutaredoxin (GLRX), and glutamate-cysteine ligase catalytic subunit (GCLC) together with energy metabolism enzymes such as glucose-6-phosphate dehydrogenase (G6PDX) and pyruvate kinase (PKLR). Also downregulated is MYH13, a sarcomeric myosin heavy chain component.

**Figure 5:**
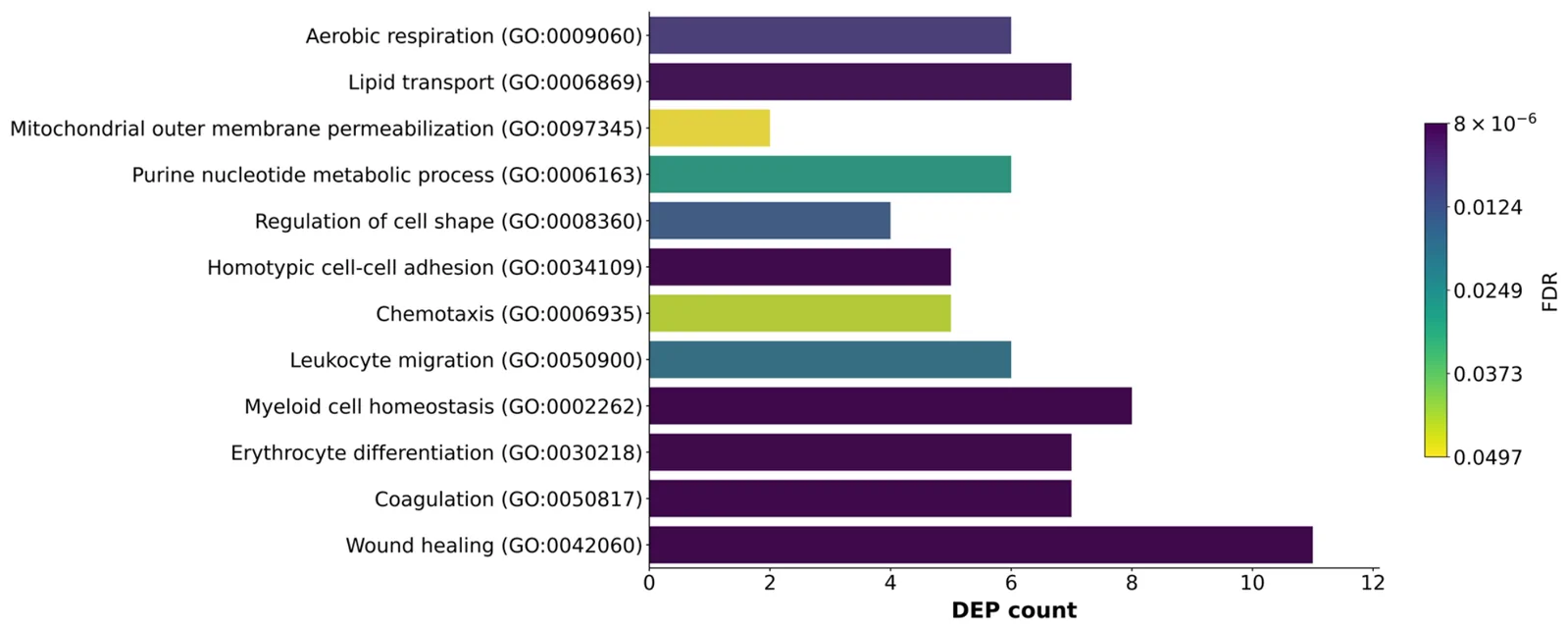
Select enriched GO terms for biological processes from ORA of on-orbit flight relative to HGC; N = 7/group. DEP count refers to the number of DEPs that matched with the GO term. Refer to Table S4 for the full list of significant terms.

ORA of differentially expressed proteins (DEPs) revealed overrepresentation of pathways associated with energy metabolism, including aerobic respiration and lipid transport, consistent with GSEA findings (Figure 5, Table S4). Additional enriched processes included mitochondrial outer membrane permeabilization, cell migration and adhesion (homotypic cell–cell adhesion, leukocyte migration, and chemotaxis), and regulation of cell shape. GO terms related to coagulation and erythropoiesis were also enriched while immune-related processes, including phagocytosis (Table S4) and myeloid cell homeostasis (Table S4, Figure 5), were also identified.

To evaluate the robustness of the ORA findings, we conducted the same analyses using alternative log_2_ FC thresholds and found that terms associated with energy metabolism such as aerobic respiration persist through the log_2_ FC threshold of |0.6|. Further, new CVD-related terms such as dilated cardiomyopathy emerge when the log_2_ FC threshold is raised to |0.7| (Tables S5–S8).

### 2.3. Cardiac Proteomic Signature During Recovery from Spaceflight

To gain insight into the molecular signals that underlie recovery from spaceflight, GSEA was conducted on the hearts of LAR groups. There were 293 significantly enriched gene sets in the LAR flight vs. HGC group (summarized into categories in Table 2, full list in Table S9). Of these significantly enriched gene sets, the greatest number were involved in cell cycle, DNA damage response, and chromosome dynamics (43 gene sets), energy metabolism and mitochondrial bioenergetics (33 gene sets), muscle contraction and contractile function (32 gene sets), and regulation of gene expression (32 gene sets) (Table 2 and Table S9). However, gene sets classified into regulation of gene expression were predominantly comprised of RPL proteins, similar to GSEA findings from the on-orbit comparisons. Likewise, most of the cell cycle and DNA damage response-associated gene sets were primarily composed of proteasome 26S subunit, non-ATPase (PSMs) family of proteins (Table 2 and Table S9). As such, the number of gene sets within these categories could be an overestimation of their enrichment in the current comparison.

Gene sets associated with cell cycle, DNA damage response, and chromosome dynamics include DNA replication (NES: 1.74) and chromosome organization (NES: −2.06) (Table S9). Some leading edge proteins for DNA replication included replication/cell-cycle regulators PCNA and CDK2, together with CDC23, an APC/C subunit involved in cell-cycle proteolysis. Leading edge proteins for chromosome organization include linker histone proteins (H1 variants), topoisomerase II beta (TOP2B) and other DNA binding proteins such BANF1 and CBX5 (Table S9).

**Figure 6:**
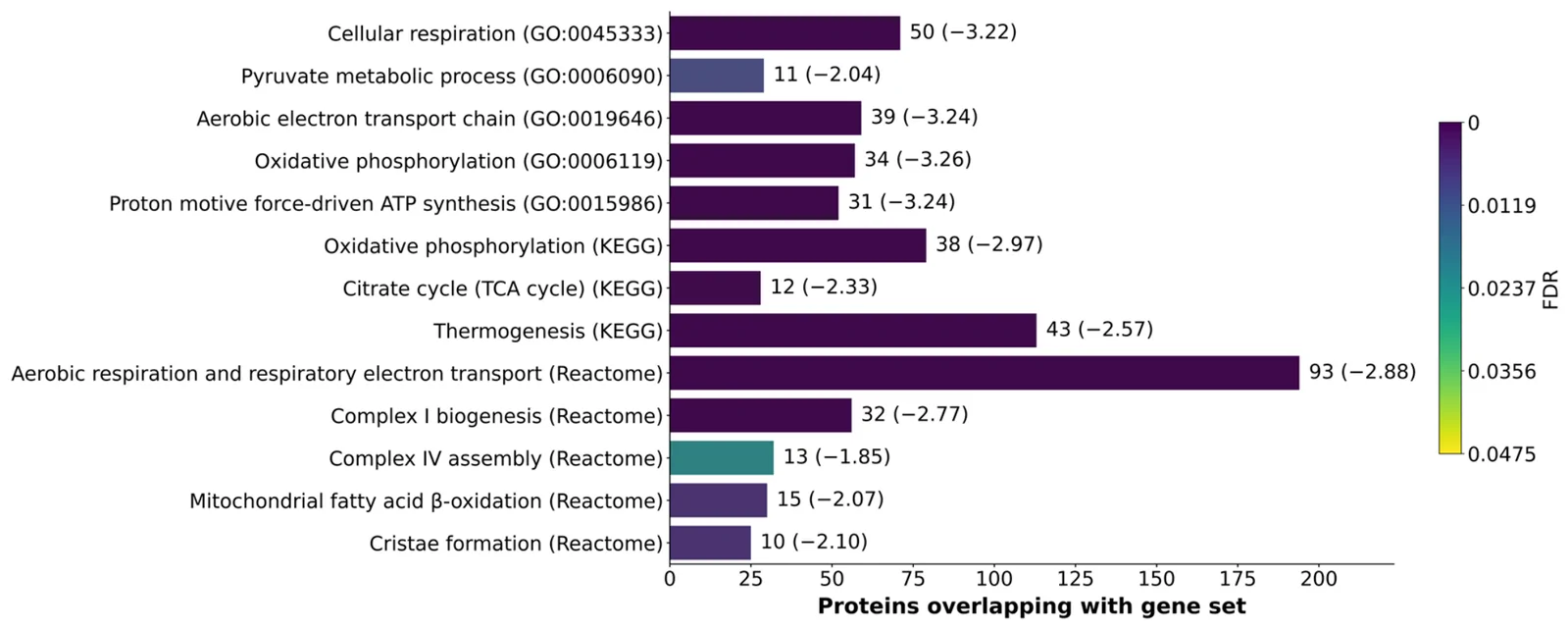
Select significant gene sets involved in energy metabolism and mitochondrial bioenergetics, respiratory chain assembly (Complex I and Complex IV), and mitochondrial organization (cristae formation) from GSEA of LAR flight vs. HGC (FDR < 0.05). The visualization scheme is as described in Figure 2. Refer to Table S9 for a full list of GSEA gene sets meeting the FDR threshold.

**Figure 7:**
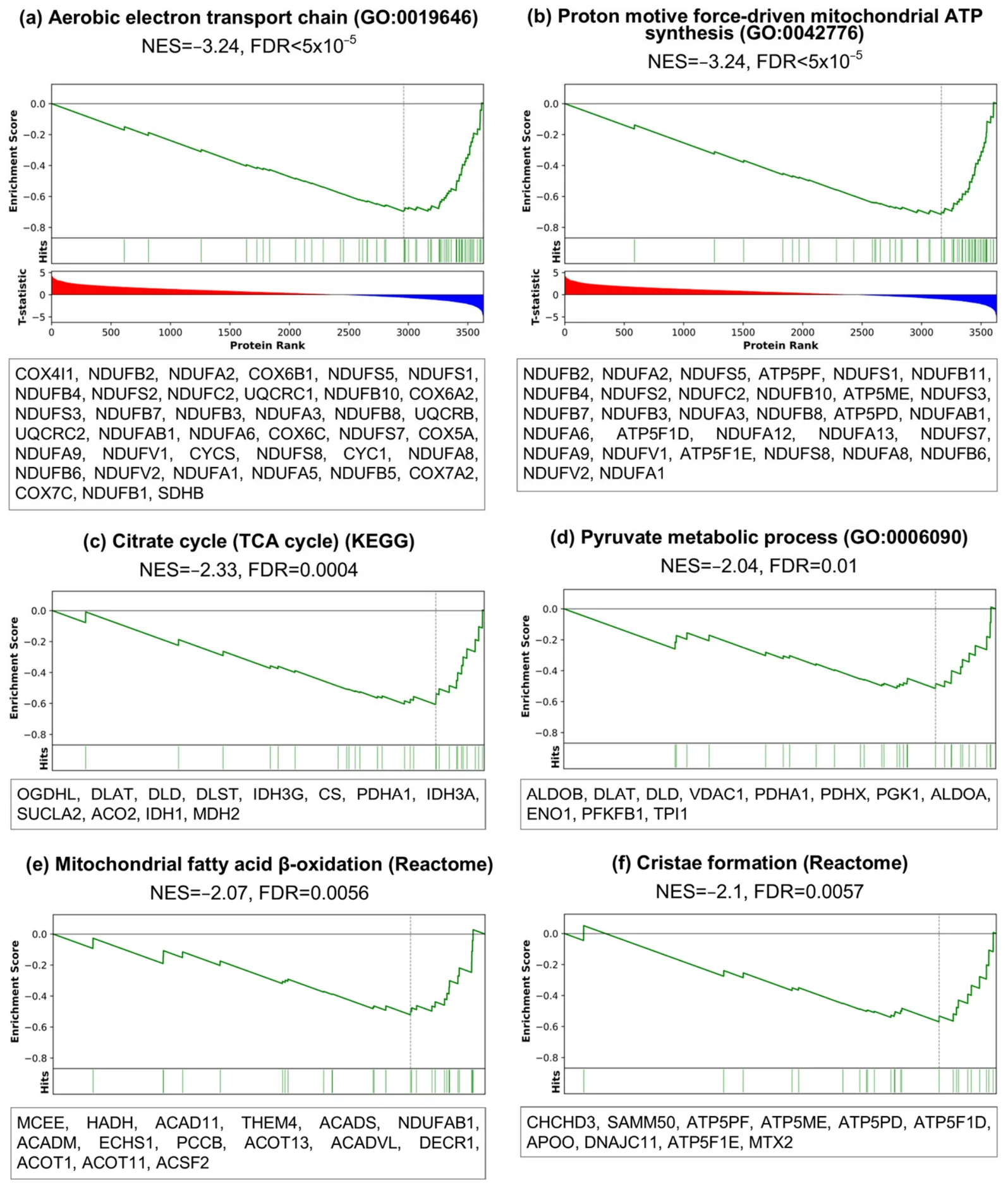
Select GSEA enrichment plot for terms associated with (**a**–**e**) energy metabolism and mitochondrial bioenergetics and (**f**) mitochondrial organization of LAR flight versus HGC. The visualization scheme is as described in Figure 3.

Similar to the on-orbit results, the LAR comparison showed significant enrichment of gene sets related to energy metabolism and mitochondrial bioenergetics. A number of these gene sets were shared between the two comparisons but enriched in opposite directions, with negative NES in the LAR comparison indicating enrichment toward the HGC group relative to flight (Figure 6 and Figure 7). Shared gene sets included proton motive force-driven mitochondrial ATP synthesis, oxidative phosphorylation, aerobic electron transport chain, cellular respiration, TCA and thermogenesis (NES: −3.26 to −2.33). Pyruvate metabolic process (NES: −2.04) and mitochondrial fatty acid β-oxidation (NES: −2.07) were unique to the LAR comparison (Figure 7).

Gene sets involved in respiratory chain assembly were also negatively enriched, including Complex I biogenesis (NES; −2.77), and Complex IV assembly (NES: −1.85), as well as mitochondrial organization, represented by cristae formation (NES: −2.1) and outer mitochondrial membrane organization (NES: −2.19) (Figure 6, Table S9). Enrichment of these pathways was driven by many of the same leading edge proteins as in the on-orbit comparisons, including NDUF proteins and mitochondrial ATP synthase (Complex V) subunits for proton motive force-driven ATP synthesis, CS and IDH for the TCA, and MICOS complex components for cristae formation (Figure 7).

Leading edge proteins for the pyruvate metabolic process included upstream glycolytic enzymes that support pyruvate production (ALDOA, ALDOB, TPI1, and PGK1), pyruvate dehydrogenase complex components, including DLAT, DLD, and PDH subunits, which catalyze the conversion of pyruvate to acetyl-CoA and thereby link glycolysis to the TCA, and VDAC1, which forms a major channel for metabolite exchange (including ATP and ADP) across the mitochondrial outer membrane (Figure 7). For mitochondrial fatty acid β-oxidation, leading edge proteins included acyl-CoA dehydrogenases (ACADs), which catalyze the initial dehydrogenation step of β-oxidation; ECHS1 and HADH, which mediate subsequent reactions in β-oxidation; and DECR1, an auxiliary enzyme required for the oxidation of unsaturated fatty acids. Also among the leading edge proteins was NDUFAB1, a component of Complex I that also functions as a mitochondrial acyl carrier protein, as well as ACOT family thioesterases and ACSF2, which contribute to acyl-CoA metabolism and homeostasis (Figure 7).

**Figure 8:**
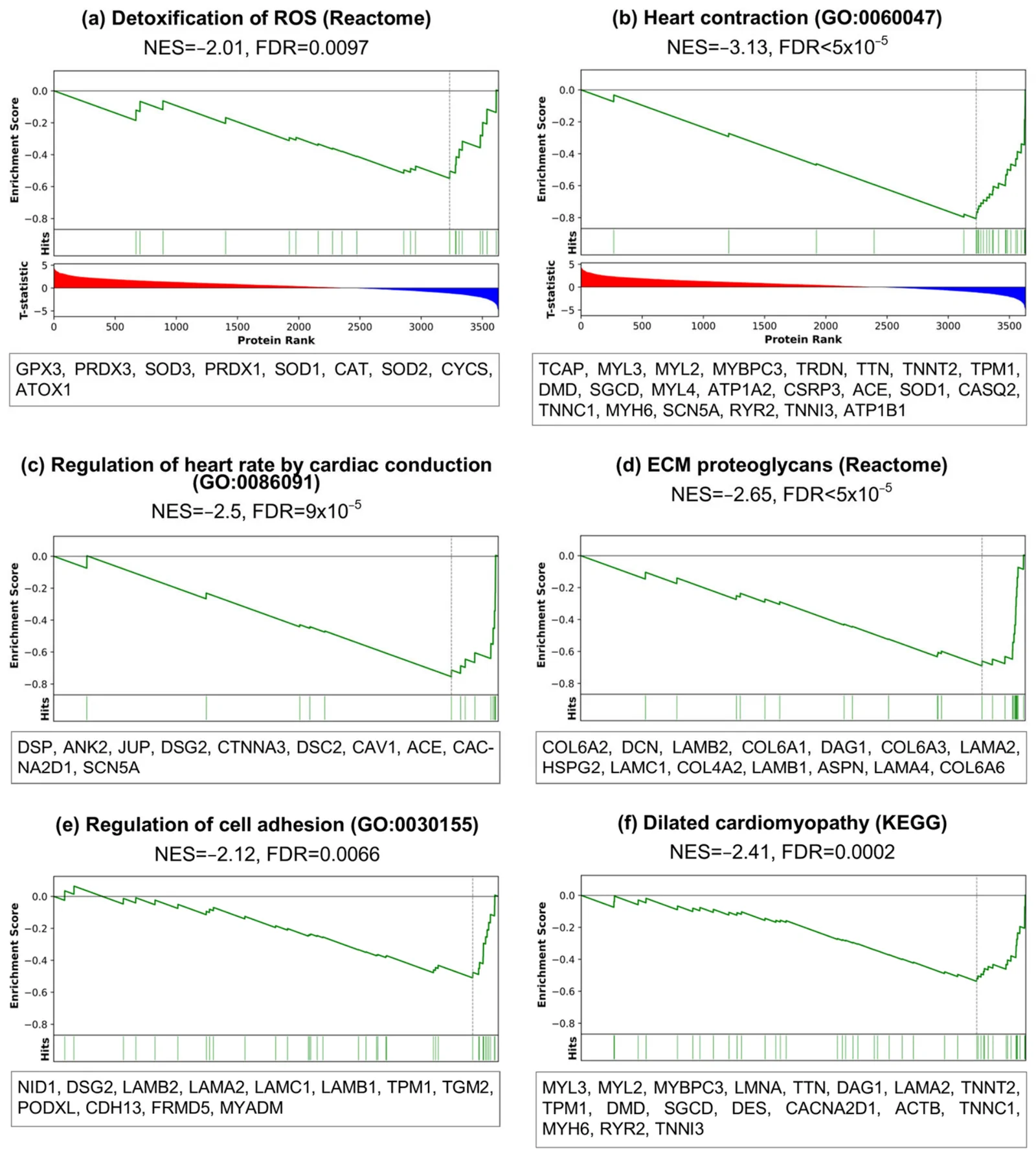
Select GSEA enrichment plot for terms associated with (**a**) redox signaling, (**b**,**c**) muscle contraction and contractile function, (**d**) cytoskeleton, cell shape and cell organization, (**e**) cell migration, chemotaxis, substrate adhesion and (**f**) cardiovascular disease of LAR flight vs. HGC. The visualization scheme is as described in Figure 3.

Notably, gene sets related to reactive oxygen species (ROS) metabolism and redox homeostasis were negatively enriched in the LAR comparison, represented by detoxification of ROS (NES: −2.01) (Figure 8a) and hydrogen peroxide metabolic process (NES: −1.88) (Table S9). Leading edge proteins included ROS detoxification enzymes such as the superoxide dismutases (SOD1, SOD2, and SOD3), CAT, GPX3, and peroxiredoxins (PRDX1 and PRDX3).

In addition, GSEA revealed negative enrichment of gene sets related to cardiac structure, contractility, ECM organization, and cell adhesion (NES: −3.13 to −2.12) (Figure 8b–e). For regulation of heart rate by cardiac conduction, leading edge proteins included desmosome-associated proteins DSP, JUP, DSG2, and DSC2, catenin alpha-3 (CTNNA3), membrane and signaling proteins ANK2 and caveolin-1 (CAV1), the cardiac sodium channel α-subunit SCN5A, the L-type calcium channel auxiliary subunit CACNA2D1, and angiotensin-converting enzyme (ACE), which plays a key role in blood pressure regulation.

Leading edge proteins for heart contraction included myosins (MYLs and MYHs), myosin-binding protein C (MYBPC3), titin (TTN), titin-cap/telethonin (TCAP), tropomyosin-1 (TPM1), and troponin complex subunits (TNNT2, TNNI3, and TNNC1), calcium-handling proteins TRDN, CASQ2, and RYR2; Na^+^/K^+^-ATPase subunits ATP1A2 and ATP1B1, the structural protein DMD, and ACE (Figure 8b,c).

Cardiovascular disease gene sets were also negatively enriched. The dilated cardiomyopathy (DCM) pathway (NES: −2.41) shared many sarcomeric and calcium-handling proteins with the heart contraction gene set, with LMNA, DAG1, DES, and ACTB additionally contributing to the DCM leading edge (Figure 8f). Leading edge proteins for ECM proteoglycans and regulation of cell adhesion included collagens, laminins, and the proteoglycans DCN, HSPG2, and ASPN, together with the basement-membrane protein NID1 and adhesion- or membrane-associated proteins DAG1, DSG2, TGM2, PODXL, CDH13, FRMD5, and MYADM (Figure 8d,e).

ORA was not conducted for the LAR comparisons because no proteins met the predefined log_2_ FC and adjusted *p*-value thresholds for differential expression (Table S2).

A summary of the proteomics findings is provided in Table S10.

### 2.4. Mitochondrial and Telomere Dynamics

**Figure 9:**
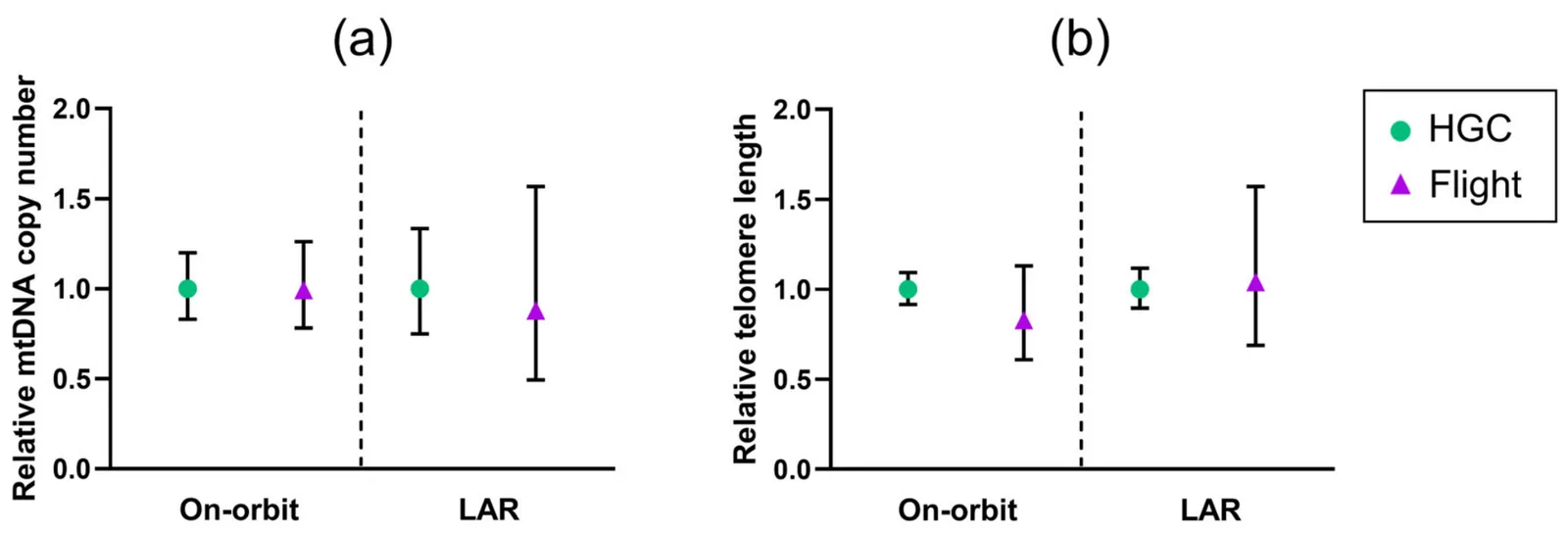
(**a**) Relative mitochondrial (mt) DNA copy numbers and (**b**) relative telomere lengths in hearts of on-orbit and LAR mice compared to corresponding HGCs, as assessed by qPCR. Values shown are means while error bars depict 95% confidence intervals. On-orbit: N = 9−10/group; LAR: N = 4/group. Within each study phase (on-orbit and LAR), the flight group was compared with its corresponding HGC. The dashed line separates the two sets of paired comparisons. Flight vs. HGC comparisons were not significant at *p* < 0.05 by Welch’s *t*-test. Aoki’s effect size, e [37], is as follows: relative mtDNA, on-orbit (0.0293) and LAR (0.3575); relative telomere length, on-orbit (0.5754) and LAR (−0.1660).

Cardiovascular diseases are associated with aging-related alterations in cellular homeostasis and mitochondrial biology [36]. We therefore assessed telomere length (via telomere copy number) and mitochondrial DNA copy number to gain insight into potential changes in telomere maintenance, cellular aging responses, and mitochondrial dynamics following spaceflight. No statistically significant differences were observed in either measurement between flight and control groups (Figure 9).

## 3. Discussion

Our findings show that spaceflight is associated with coordinated remodeling of the cardiac proteome involving mitochondrial bioenergetics, substrate metabolism, and redox homeostasis, in support of our hypothesis and consistent with prior results from other experimental models. Hearts from female mice that were collected ~3H after return to Earth during the STS-131 mission, showed upregulation of oxidative stress-related genes [22]. Whereas space-flown human-induced pluripotent cell (iPSC)-derived cardiomyocytes displayed upregulated mitochondrial bioenergetics-related pathways, including aerobic respiration and electron transport chain processes [38]. Similarly, human iPSC-derived cardiomyocytes integrated into a heart-on-a-chip platform flown on the ISS also exhibited altered expression of genes involved in the oxidative stress response and mitochondrial metabolism, together with impairments in force production [39].

In the current study, spaceflight induced positive enrichment of energy metabolism and mitochondrial bioenergetic gene sets such as oxidative phosphorylation (OXPHOS), cellular respiration, TCA, and respiratory chain complex assembly in the cardiac proteome. This is corroborated at the individual protein level where leading edge and upregulated DEPs included multiple ETC subunits, mitochondrial complex assembly factors, and the mitochondrial pyruvate carrier MPC1. The increased abundance of mitochondrial and OXPHOS proteins may reflect increased OXPHOS capacity. However, the increase in ETC and TCA components may not necessarily result in enhanced enzymatic activity or ATP output; the results may instead reflect a compensatory mechanism to overcome any OXPHOS inefficiencies and/or deficits in ATP output.

In addition to changes in mitochondrial bioenergetic pathways, the proteomic signature suggests altered glucose metabolism during spaceflight. Although the adult mammalian heart relies predominantly on mitochondrial oxidative phosphorylation to meet ATP demands, glycolytic activity remains important for substrate flexibility, redox balance, and metabolic signaling [40]. The observed downregulation of PKLR, which catalyzes the terminal step of glycolysis to generate pyruvate, together with reduced expression of the glucose transporter SLC2A3, may indicate reduced glucose uptake and glycolytic flux. Conversely, increased MPC1 abundance may represent a compensatory response to preserve mitochondrial pyruvate utilization capacity despite potential reductions in glycolytic pyruvate production. These findings are consistent with reported impairments in myocardial glucose utilization and decreased pyruvate dehydrogenase activity in rats that underwent HU [41]. Similarly, another study showed that left ventricular glucose uptake decreases during early HU and precedes impairments in cardiac structure and function [42]. Together, these observations raise the possibility that impaired glucose utilization contributes to insufficient metabolic support for myocardial function during spaceflight.

Further, MICOS10 and CHCHD10, proteins that regulate cristae morphology and mitochondrial membrane organization, showed increased abundance, suggesting coordinated remodeling of mitochondrial architecture and mitochondrial ATP production pathways during spaceflight. Notably, this protein-level remodeling occurred without a corresponding change in mitochondrial DNA copy number during spaceflight. Space-flown human iPSC-derived cardiomyocytes exhibit a more fragmented mitochondrial architecture compared to ground controls [39]. However, mitochondrial architecture and mtDNA integrity were not assessed in the current study, precluding direct assessment of whether the observed changes in cristae-associated proteins were accompanied by structural or genomic alterations. Similarly, mitochondrial DNA copy number did not differ significantly between flight and control groups during recovery, although these findings should be interpreted cautiously given the small sample sizes of the recovery groups.

Many of the energy metabolism and mitochondrial pathways enriched during flight also were found during recovery but in the opposite direction (i.e., negative; proteins and processes enriched towards controls), examples of which include OXPHOS, cellular respiration, and proton motive force-driven ATP synthesis. Although some leading edge proteins were shared between the two comparisons, others were unique, indicating that the molecular programs underlying these pathways differ between flight and recovery. It is unclear whether these molecular signatures from recovery reflect the establishment of a distinct postflight metabolic state. Because functional and other biochemical assessments of proteins involved in mitochondrial respiration, ATP production, and ROS generation were not performed in this study, the metabolic consequences of this proteomic remodeling, both during flight and recovery, could not be directly established and merit further investigation.

During spaceflight, increases in OXPHOS protein abundance occurred alongside changes in the antioxidant defense cascade. CAT, GPX1, GLRX, GCLC, and G6PD were all downregulated, while SOD2 was modestly upregulated. SOD2 serves as the primary antioxidant defense within the mitochondrial matrix, converting superoxide generated by ETC electron leak into hydrogen peroxide; hence, its upregulation together with increased OXPHOS protein abundance is expected. However, antioxidant enzymes downstream of SOD2 that participate in clearing hydrogen peroxide were downregulated, including CAT, GPX1, G6PD, GLRX, and GCLC. Hydrogen peroxide is subsequently decomposed by CAT and GPX1 [43]. GPX1 utilizes reduced glutathione (GSH), whose synthesis requires GCLC and whose redox cycling is supported by NADPH generated primarily by G6PD, the rate-limiting enzyme of the pentose phosphate pathway. GLRX further contributes to cellular redox homeostasis by using GSH to reverse oxidative modifications of protein thiols [44,45]. Collectively, the coordinated downregulation of these antioxidant proteins suggests a reduced capacity to clear hydrogen peroxide, potentially creating a bottleneck in antioxidant defense that promotes oxidative damage. These findings suggest that prolonged spaceflight may result in a molecular signature that renders the heart more susceptible to oxidative stress, which warrants further investigation given the reports of elevated biomarkers for oxidative stress during flight in ISS crew members [46], and the higher levels of ionizing radiation anticipated during deep space missions. Together, our results are consistent with evidence from other animal models of spaceflight and ground-based simulations, suggesting that spaceflight can perturb mitochondrial ROS signaling and redox balance [21,22,47,48,49,50,51]. Enhanced mitochondrial ROS quenching via targeted overexpression of catalase (MCAT) mitigate neuroinflammatory changes induced by HU [47,48]. In other animal studies, treatment with a MnSOD (SOD2) mimetic ameliorated the negative effects of simulated microgravity across multiple tissues such as marrow, brain and retina [49,50,51,52]. On the other hand, the plasma from ISS crew members show increased cell-free mitochondrial DNA and RNA during flight [53]. Together, these findings provide a rationale for targeting the mitochondria and its ROS-quenching pathways as a countermeasure strategy to mitigate spaceflight-induced tissue dysfunction.

The recovery phase also showed enrichment of redox signaling pathways specifically, negative enrichment of the hydrogen peroxide metabolic process and positive enrichment of the KEAP1-NFE2L2 pathway, a critical regulator of the cellular response to oxidative stress [54]. However, the changes in expression of key antioxidants were modest compared to those observed in the on-orbit comparisons. Regardless, these findings suggest that the heart adapts to Earth conditions upon return by remodeling antioxidant signaling pathways, consistent with findings from hearts of STS-131 female mice shortly after return to Earth [22]. Like other outcomes from the LAR cohort, proteomic findings are based on a limited sample size. Hence, the GSEA results and the absence of DEPs should be interpreted cautiously.

The overall increase in the abundance of cytosolic ribosomal proteins (RPLs) during spaceflight reflects a biologically meaningful signal, despite the likely overestimation of enriched translation-related processes. This expression pattern contrasted with that of mitochondrial ribosomal proteins (MRPs) which generally decreased during flight. Together, these findings suggest that spaceflight alters cardiac translational homeostasis with divergent regulation of cytosolic versus mitochondrial protein synthesis machinery. An increase in the ETC/OXPHOS protein abundance occurs together with an overall reduction in the expression of MRPs. These two observations may appear contradictory, since mitochondrial ribosomes are required to translate the 13 mtDNA-encoded ETC subunits. However, mitochondria-encoded ETC proteins can remain abundant, reflecting prior synthesis and the relatively slow turnover of assembled respiratory complexes [55]. The proteomic signature of recovery also reflects remodeling of the translational machinery that is distinct from that of spaceflight.

During spaceflight, proteins involved in muscle contraction were also differentially expressed although few enriched gene sets emerged. In contrast, recovery was associated with negative enrichment of multiple gene sets involved in sarcomere organization, heart rate regulation, and contractile function, consistent with adaptations to hemodynamic and gravitational loading changes upon return to Earth. Notably, proteins associated with ECM remodeling and cardiovascular disease (dilated cardiomyopathies) were also negatively enriched during recovery, consistent with other analog studies [22,56,57]. These findings suggest that the recovery period represents a dynamic phase of cardiac adaptation that may influence susceptibility to cardiovascular dysfunction following spaceflight. Our findings differ in some respects from those of NASA RR-23, which examined male mice flown to the ISS for 38.5 days and euthanized within 18 h of the return to Earth. In that study, gene ontology analysis showed no changes in genes associated with sarcomere contractility between flight and ground control groups [23]. Several experimental factors differ between RR-23 and the present study, including age at launch, and the time between return to Earth and tissue collection. These differences in experimental design may contribute to the distinct molecular profiles observed in the two studies. Consequently, the relative contributions of sex and postflight recovery to cardiac contractile pathways and other molecular responses cannot be readily distinguished.

Spaceflight also resulted in downregulation of platelet and coagulation-associated proteins, erythrocyte-associated proteins (e.g., hemoglobins) and innate immune system components such as complement proteins which may reflect changes in the composition of blood retained within the ventricular chamber and coronary microvasculature at the time of tissue collection. These patterns of expression are consistent with the observation of anemia in space mission crew members [58,59]. Additionally, the expression patterns could also reflect downregulation of aspects of immune function, consistent with reports of immune dysfunction in ISS crew [60]. The recovery phase also showed enrichment of immune-related pathways, although the changes in expression of key proteins were modest compared to those observed during spaceflight.

Spaceflight and recovery elicited distinct patterns of proteomic remodeling. During spaceflight, substantial changes in individual proteins yielded numerous DEPs for ORA but relatively few significantly enriched gene sets by GSEA. This suggests that the adaptation to extended spaceflight is mostly driven by a smaller subset of molecular nodes. Because only a single in-flight time point was examined, it remains unclear whether this pattern extends to earlier or later phases of spaceflight. In contrast, recovery was characterized by modest changes in individual proteins that did not meet DEP thresholds, yet many functionally related proteins shifted concordantly, resulting in a greater number of enriched pathways. Together, these findings indicate that cardiac recovery is not simply a reversal of in-flight molecular changes but instead reflects a more distributed, systems-level reorganization of the cardiac proteome.

An intriguing aspect of this current study is that the LAR animals are from the same cohort included in a study reporting transgenerational effects of spaceflight [27]. In the prior report, although returned females remained fertile with a modest reduction in fecundity, their offspring exhibited deficits in growth, metabolism, muscle force production, behavior, and reproductive capacity that extended into the F2 generation, suggesting persistent effects of maternal spaceflight exposure. Whether spaceflight has generational impacts on cardiac metabolism and overall cardiovascular health is unclear and merits further study.

In summary, spaceflight induces remodeling of the female cardiac proteome, involving mitochondrial bioenergetics, redox regulation, translational processes, and pathways associated with cardiac structure and function during both flight and recovery. The distinct molecular signatures observed across these phases highlight the dynamic nature of cardiac adaptation to the spaceflight environment and subsequent physiological transition after the return to Earth. These findings provide insight into potential mechanisms underlying cardiovascular risk associated with spaceflight, including altered mitochondrial function, oxidative stress vulnerability, and remodeling of contractile and ECM pathways.

## 4. Materials and Methods

Overall design of spaceflight study: Preflight acclimation of animals are described in [27]. Forty females at 11 weeks of age were launched on SpaceX Commercial Resupply Service (CRS)-29 and housed onboard the ISS for 42−60 days as part of NASA RR-20 using a previously described NASA rodent habitat [61] with a nesting hut [62]. Mice were maintained in a 12:12 h light:dark cycle with lights on/off at 0700/1900 GMT. Of the 40 mice that inhabited the ISS, 20 were assigned to the “on-orbit” group while the remaining 20 were part of the “live animal return” (LAR) group. The on-orbit groups were the animals euthanized on board the ISS (on-orbit flight) and their corresponding habitat and age-matched control group on Earth (on-orbit HGC). The LAR groups included animals that returned to Earth (LAR flight) and euthanized thereafter and their corresponding habitat, and age-matched ground control (LAR HGC). Both on-orbit and LAR HGC groups were maintained on Earth in the ISS environmental simulator (ISSES) chamber (details provided below). Refer to Supplementary Methods for additional details on animal experiments, determination of sample sizes and randomization strategies.

On-orbit groups: To stimulate follicular development, the on-orbit flight mice received an intraperitoneal (IP) injection of pregnant mare serum gonadotropin (PMSG) aboard the ISS between flight days 55−60. At 46−48 h post-injection, five mice were euthanized while the remaining 15 were administered human chorionic gonadotropin (hCG) via the IP route to induce ovulation [63,64]. This was done since one of the primary aims of the NASA RR-20 study is to determine the effect of spaceflight on female reproductive health [27]. Sixteen hours after hCG injection, euthanasia was conducted by ketamine/xylazine administration, with terminal blood drawn by closed cardiac puncture, followed by bilateral thoracotomy. Carcasses with hearts intact were wrapped in aluminum foil and frozen at −185 °C using the ISS rapid freeze hardware and stored in −80 °C until the sample returned to Earth. The current study analyzed a subset of the 15 on-orbit flight animals that received both PMSG and hCG injections. The frozen carcasses of the on-orbit flight group were transported to Earth at cryogenic temperatures and then shipped on dry ice to KUMC for further dissection. At the time of dissection, the carcass was thawed once, kept on ice during tissue dissection, and hearts collected shortly thereafter.

The corresponding 20 HGC mice of the on-orbit flight group were housed at the Roskamp Institute in Sarasota, Florida, using the same model of rodent habitat onboard the ISS. The habitat was maintained at 28 to 29 °C, within the ISS environmental simulator (ISSES) chamber which mimics ISS conditions minus microgravity [27]. The HGC mice were maintained at a five-day offset to enable replication of ISS environmental conditions. Euthanasia procedure and the handling of HGC carcasses were similar to those of the flight group.

LAR groups: Twenty mice designated as the LAR flight group were returned to Earth via the SpaceX Dragon capsule, after 42 days of spaceflight. Mice were transported to Roskamp Institute within 12 h of the Dragon capsule splashdown. Of the 20 LAR flight mice, four remained on GMT time following the return to Earth. At 2.5 days post-return to Earth, these mice received PMSG and hCG injections as described in [27]. At 16 h post-hCG administration, these four LAR flight mice were euthanized and tissues were collected shortly thereafter. Similarly, the LAR HGC mice (N = 4) were euthanized five days later to account for the offset for replication of the ISS conditions in the ISSES.

Heart collection and processing: Shortly after collection, hearts were bisected into superior (consists of atria and portion of ventricles) and inferior halves. The inferior portion was further dissected to isolate the right ventricle (with interventricular septum) and left ventricle, flash frozen in liquid nitrogen, and stored at −80 °C until processing. Unlike on- orbit groups, heart collection of LAR groups was conducted shortly after euthanasia (no prior freezing of carcass).

Preparation of heart tissue for mass spectrometry: Left ventricles from the inferior half of the heart were used for proteomic analysis. Tissues were homogenized in RIPA buffer with protease and phosphatase inhibitors (all from Thermo Fisher Scientific, Waltham, MA, USA) followed by sonication. Lysates were clarified by centrifugation at 10,000 rpm for 10 min at 4 °C and supernatants were recovered.

Protein digestion for liquid chromatography and mass spectrometry (LC-MS): Protein concentration was determined using the Protein Assay EZQ (Invitrogen, Waltham, MA, USA). For each sample, 100 μg of protein extract was reduced, alkylated with iodoacetamide, and enzymatically digested using trypsin/Lys-C with the EasyPep Mini MS Sample Prep Kit (Thermo Scientific, Waltham, MA, USA) at an enzyme:protein ratio 1:10. Peptides were column-purified and reconstituted in 0.1% formic acid to a final concentration of 1 μg/μL for LC-MS.

Liquid chromatography and mass spectrometry: Peptide analysis was performed with an Evosep One LC system coupled to a Bruker timsTOF Pro 2 mass spectrometer using data-independent acquisition parallel accumulation-serial fragmentation (DIA-PASEF) [65,66,67]. Peptides were separated using a 30 samples/day method on a PepSep C18 column (15 cm × 150 μm, 1.5 μm packing) heated to 50 °C. Separated peptides were then ionized using CaptiveSpray (Bruker, Billerica, MA, USA) and MS analysis performed on a timsTOF Pro2 mass spectrometer using DIA-PASEF with MS1 scan range from 100 to 1700 m/z. MS2 was performed using a staggered window scheme over 300 to 1200 m/z utilizing 7 MS/MS ramps and 25 MS/MS windows with mobility range 0.70 to 1.30 1/K0. A ramp time of 85 ms was set, allowing for a minimum of seven points across the peak. Data was analyzed using Spectronaut v19.1.240806 (Biognosys, Newton, MA, USA) and DirectDIA against UniProt mouse reference proteome (UP000000589) containing 55,286 sequences and known potential contaminants (Crap_uniprot_with_human_MRSonbeadV2) containing 117 sequences. Protein label-free quantification (LFQ) and cross-normalization were set to automatic, with Spectronaut applying MaxLFQ and global normalization, respectively. Post-translational modification (PTM) analysis settings were as follows. Carbamidomethyl (C) was set as fixed modification while acetyl (protein N-term), oxidation (M), dioxidation and trioxidation were set as variable modifications.

Post-MS processing and quality control: Normalized protein quantities and precursor counts were exported from Spectronaut v20.3 using the original Spectronaut v19 analysis file. Protein annotations were curated by removing non-mouse proteins and redundant protein entries. Protein intensities were imported into Rstudio (2025.09.2 Build 418), converted to log_2_ scale, and filtered to retain the proteins quantified in at least 50% of samples within at least one experimental group. Quality control (QC) included sample boxplots, *p*-value distributions, and PCA using the NIPALS algorithm with unit-variance scaling. Missing values were retained as “NA” for downstream analyses but replaced with zero for PCA visualization only.

Statistical analysis and calculation of log_2_ fold changes: Differential expression was analyzed using a limma-DEqMS workflow [68,69,70]. Linear modeling was performed with limma (v3.68.4) using empirical Bayes variance moderation (trend = TRUE, robust = TRUE), and DEqMS (v1.30.0) was used to model protein-specific variance based on precursor counts. Per-protein precursor counts were summarized as the minimum across samples. No imputation was performed prior to differential expression analysis. Outputs included log_2_ fold changes, moderated t-statistics, raw *p*-values, Benjamini–Hochberg (BH) adjusted *p*-values [71], and Storey q-values [72,73].

Gene set enrichment analysis: GSEA was performed using GSEApy (v1.3.0) [74,75]. Proteins were ranked by the DEqMS moderated t-statistic and analyzed using 2000 gene set permutations against Enrichr [76] gene set library GO biological process 2026, KEGG 2019 mouse, and Reactome pathways 2024. Gene sets containing 10−300 genes were included in the analysis. Pathways with FDR-adjusted *p* < 0.05 were considered significantly enriched.

Overrepresentation analysis: DEPs were defined as those with a log_2_ fold change >0.4 or <−0.4 and a Storey q-value < 0.05 (Refer to Supplementary Methods and Figure S3 for further details on Storey q-values). We selected |log_2_ FC| > 0.4 for ORA to capture coordinated changes of modest magnitude among proteins participating in the same biological processes. Functional enrichment analysis was performed using gProfiler [77], with gene set sizes of 10−300, BH-adjusted *p* < 0.05, and exclusion of electronically inferred annotations. Enriched GO biological process terms and KEGG pathways were identified.

Telomere length and mitochondrial DNA copy number assays: DNA was isolated from right ventricular tissue using the DNeasy Blood and Tissue Kit (Qiagen, Germantown, MD, USA). Quantitative PCR (qPCR) was performed using 1 ng DNA in a 20 µL reaction volume. Relative telomere length was measured using the Mouse Relative Telomere Quantification Assay Kit (ScienCell, Carlsbad, CA, USA). Mitochondrial DNA copy number was determined using Luna Universal qPCR Master Mix (New England Biolabs, Ipswich, MA, USA) with previously described mouse hexokinase and mitochondrial NADH dehydrogenase subunit 1 (ND1) primers [78]. Cycling conditions and primer sequences are provided in the Supplementary Methods.

Statistical analysis of qPCR data: Technical replicate Cq values were averaged prior to analysis. Statistical analyses were performed on ΔCt values. Normality and homogeneity of variance were assessed using the Shapiro–Wilk and Brown–Forsythe tests, respectively. Because unequal variances were observed for some comparisons, Welch’s *t*-test was used throughout (JMP Student Edition v18.2.2). Relative fold changes were determined using the ΔΔCt method [79,80], and 95% confidence intervals were calculated. Effect size, e, was calculated using Aoki’s method [37], which does not assume equal variances.

## Figures and Tables

**Table 1:** Summary of enriched biological process gene ontology (GO) terms, KEGG and Reactome pathways by category from GSEA of on-orbit flight vs. HGC group (Total: 67 gene sets). N = 7/group. Refer to Table S3 for a full list of significant enriched gene sets.

Category	GO Term	KEGG	Reactome	Total
Regulation of gene expression	3	1	21	25
Energy metabolism and mitochondrial bioenergetics	8	3	2	13
Other disease (non-CVD)	0	4	4	8
Other metabolic process	3	0	5	8
Muscle contraction and contractile function	4	0	0	4
Immune regulation and response	0	1	0	1
Material transport	0	0	1	1
Mitochondrial organization	0	0	1	1
Respiratory chain assembly	0	0	2	2
Other signal transduction	0	1	2	3
Other, miscellaneous	0	0	1	1

**Table 2:** Number of enriched biological process GO terms, KEGG and Reactome pathways by category from GSEA of LAR flight vs. HGC group. N = 4/group. Refer to Table S9 for a full list of significant enriched gene sets.

Category	GO Term	KEGG	Reactome	Total
Cell cycle, DNA damage response, and chromosome dynamics	4	0	39	43
Energy metabolism and mitochondrial bioenergetics	20	6	7	33
Muscle contraction and contractile function	29	1	2	32
Regulation of gene expression	2	0	30	32
Material transport	3	2	17	22
Immune regulation and response	0	0	21	21
Other disease (non-CVD)	0	4	13	17
Protein folding and degradation	0	0	13	13
Cell migration, chemotaxis, substrate adhesion	5	1	4	10
Cytoskeleton, cell shape and cell organization	1	0	6	7
Other metabolic process	2	0	5	7
Apoptosis/cell death	0	0	5	5
Cardiovascular disease	0	4	0	4
Respiratory chain assembly	2	0	2	4
Redox signaling	1	0	2	3
Mitochondrial organization	1	0	1	2
Peroxisome signaling	0	1	1	2
Other signal transduction	0	1	32	33
Other, miscellaneous	1	0	2	3

## Data Availability

The original contributions presented in this study are included in the article/Supplementary Materials. Further inquiries can be directed to the corresponding author.

## Supplementary Materials

The following supporting information can be downloaded at https://www.mdpi.com/article/10.3390/ijms27209215/s1.
