# Supplementary material for: Proteomic Remodeling in the Female Heart During Spaceflight and Recovery
Source: Int J Mol Sci. 2026 Oct 17;27(20):9215. doi: 10.3390/ijms27209215 (PMC13705333; doi:10.3390/ijms27209215)
Supplement: Supplementary file 1 [file ijms-27-09215-s001.zip › Figure S1. Log2FC distributions with caption.pdf]

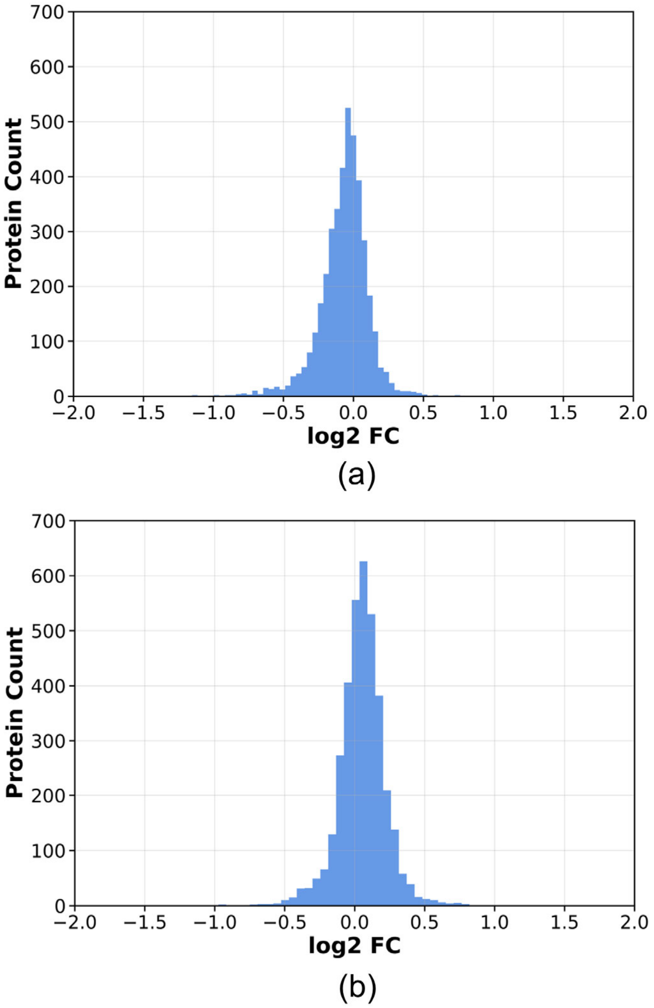

Figure S1. Log_2 FC distributions of (**a**) on orbit flight versus HGC and (**b**) LAR flight versus HGC. On orbit: N = 7/group; LAR: N = 4/group.
