# Supplementary material for: Proteomic Remodeling in the Female Heart During Spaceflight and Recovery
Source: Int J Mol Sci. 2026 Oct 17;27(20):9215. doi: 10.3390/ijms27209215 (PMC13705333; doi:10.3390/ijms27209215)
Supplement: Supplementary file 1 [file ijms-27-09215-s001.zip › Figure S2. PCA with caption.pdf]

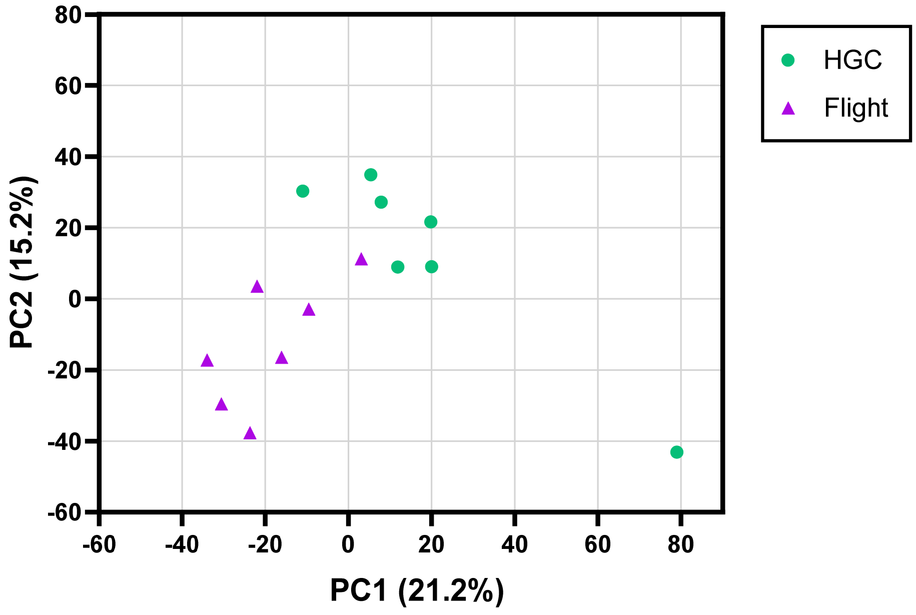

(**a**)

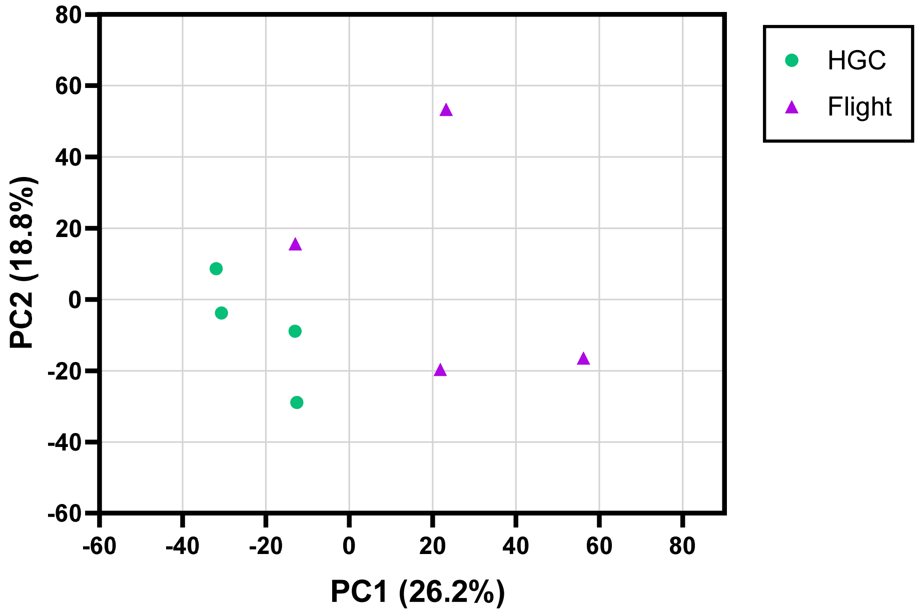

(**b**)

Figure S2. PCA of (**a**) on orbit flight versus HGC and (**b**) LAR flight versus HGC group. The numbers enclosed in parentheses represent the percentage of the total variance explained by the principal component.
