# Supplementary material for: Proteomic Remodeling in the Female Heart During Spaceflight and Recovery
Source: Int J Mol Sci. 2026 Oct 17;27(20):9215. doi: 10.3390/ijms27209215 (PMC13705333; doi:10.3390/ijms27209215)
Supplement: Supplementary file 1 [file ijms-27-09215-s001.zip › Figure S3. On Orbit p val distribution with caption.pdf]

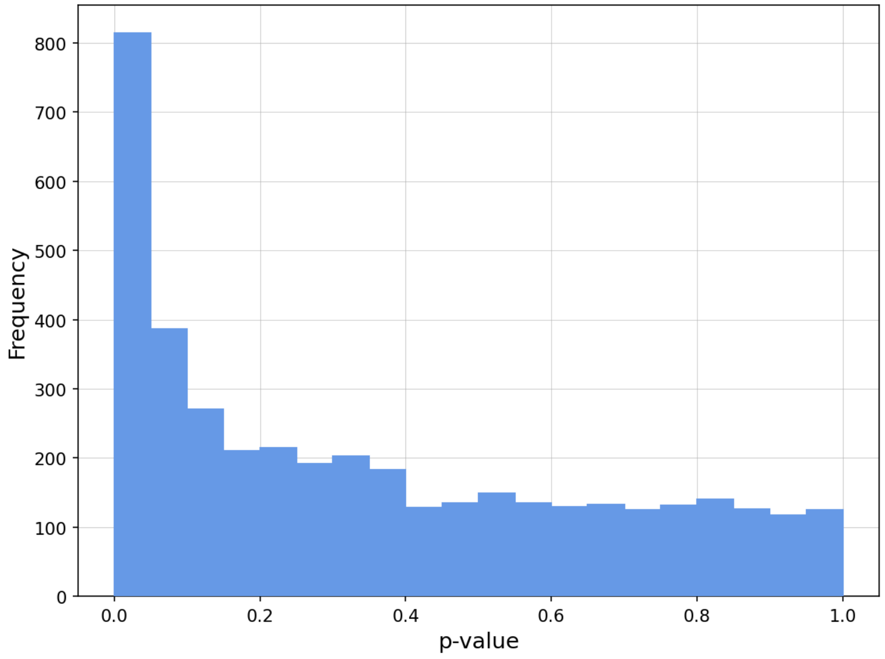

Figure S3. Unadjusted p-values of on orbit flight versus HGC group.
