# Supplementary material for: Proteomic Remodeling in the Female Heart During Spaceflight and Recovery
Source: Int J Mol Sci. 2026 Oct 17;27(20):9215. doi: 10.3390/ijms27209215 (PMC13705333; doi:10.3390/ijms27209215)
Supplement: Supplementary file 1 [file ijms-27-09215-s001.zip › Supplementary Methods.pdf]

Supplementary Methods

Determination of sample sizes and outcome measures. The total number of animals that underwent the experimental procedures described in the Methods section was determined based on the primary scientific objectives of NASA RR-20 (female fecundity) [1]. Sample sizes for the proteomics and qPCR analyses for on-orbit groups were determined separately based on prior power analysis (at least N=7 per group) and supported by prior experimental data from spaceflight rodent experiments and analogs. Sample sizes of LAR groups (N=4/group) were limited due to logistical constraints of a spaceflight experiment and are stated as a limitation in the Discussion.

Randomization and criteria for inclusion and exclusion. Animals showing evidence of barbering were excluded from the cohorts that were used in the actual NASA RR-20 experiment. To randomize and assign to experimental groups, animals were ranked based on body weight and distributed among the experimental groups to achieve similar body weight distributions across groups. All animals that were flown into space and their corresponding ground controls completed the experiment.

For heart processing and downstream assays, hearts that were not dissected according to the predefined sample collection protocol were excluded from protein lysate preparation and DNA extraction. For each on-orbit group (flight or HGC), seven of the 15 available heart samples were used for proteomics and were selected randomly. For qPCR, 9-10 samples/group were used. All available hearts from the LAR recovery groups (flight and HGC, N=4/group) were included in proteomic and qPCR analysis.

To minimize potential batch effects on experiment outcomes, flight and control carcasses were dissected alternately. For processing (protein lysate prep and DNA extraction), typically, the same number of flight and control hearts were handled on any given processing day.

All samples analyzed by LC-MS were used in downstream proteomic analysis (no datapoint was excluded). Similarly, all samples analyzed by qPCR were used in the calculation of ΔCT values and ensuing statistical analysis.

Monitoring of animals and humane endpoints. Animals were evaluated daily via video health checks; movement and coat appearance were visually examined. All animals were euthanized following follicular stimulation to collect synchronized samples.

Sample coding. Personnel involved in animal handling and sample collection were aware of flight and ground control assignments, as the source and experimental status of the animals were inherently identifiable within the spaceflight study design. Following heart collection, samples were coded for processing and downstream assays where feasible, and group identities were revealed after completion of the respective assays.

Protocol registration statement. All animal groups and procedures included in the NASA RR-20 study were pre-approved as per IACUC protocols described in the Methods section. For the proteomics and qPCR analysis of hearts, a study protocol specifying the research question, key design features, and planned analyses was established prior to conducting assays but was not formally registered or publicly available.

Use of Storey q-value for ORA of on-orbit flight versus HGC group. The Storey q-values and the DEqMS Benjamini-Hochberg (BH)-adjusted p-values are derived from the same DEqMS p-values and differ in how the false discovery rate (FDR) is estimated. The Benjamini-Hochberg procedure [2] assumes a conservative upper bound of $\pi_0 = 1$ (i.e., that all tested hypotheses may be null), whereas the Storey q-value method [3,4] estimates π_0 directly from the p-value distribution. When a substantial fraction of proteins are truly differentially expressed ($\pi_0 < 1$), estimating π_0 can yield better-calibrated FDR estimates than the BH procedure. Inspection of the unadjusted p-value distribution (Figure S3) provided initial motivation for this approach. The p-value distribution exhibited a pronounced peak near $p < 0.05$ and flattened as p approached 1, a shape consistent with a mixture of a non-null component concentrated near zero and a uniform null component across (0, 1), suggesting that a meaningful fraction of proteins (~40%, i.e., $1 - \pi_0 \approx 0.40$) depart from the null. Under these conditions, the BH procedure is unnecessarily conservative: by assuming $\pi_0 = 1$, it overestimates the proportion of null hypotheses and sacrifices discovery power. The upper tail of the plot was approximately uniform, indicating that a stable π_0 estimate was obtainable. To confirm this, three test criteria were evaluated prior to adoption. First, π_0 was estimated using two independent methods: the "smoother" ($\pi_0 = 0.603$) and bootstrap ($\pi_0 = 0.629$) method, which yielded a difference of 0.026, well within the generally accepted concordance threshold of 0.05. Second, the π_0 estimates (~0.60–0.63) are well within the boundary values (0 or 1), rather than approaching either extreme. Third, the λ vs $\pi_0(\lambda)$ diagnostic plot showed a decreasing curve that flattened into a plateau ranging from $\pi_0 \approx 0.60$ to 0.65 for $\lambda > 0.4$, with modest point-to-point variation, including a slight dip and rebound at the highest λ values, reflecting the smaller number of p-values available as λ increases. The "smoother" closely tracked the overall trend across this range. Altogether, these tests confirm that the assumptions underlying the Storey q-value are satisfied for this dataset.

Telomere length assay by qPCR. Thermocycler settings were as follows: an initial denaturation step at 95 °C for 10 min, followed by 32 cycles of denaturation at 95 °C for 20 sec, annealing at 52 °C for 20 sec, and extension at 72 °C for 45 sec.

Mitochondrial (mt) DNA copy number assay by qPCR. The following primers as previously described: ND1 Forward: CTAGCAGAAACAAACCGGGC, ND1 Reverse: CCGGCTGCGTATTCTACGTT, Hexokinase-F: GCCAGCCTCTCCTGATTTTAGTGT,

Hexokinase-R: GGGAACACAAAAGACCTCTTCTGG [5]. Thermocycler settings were as follows: initial denaturation at 95 °C for 60 sec, followed by 42 cycles of denaturation at 95°C for 15 sec and a combined annealing/extension step at 60 °C for 30 sec.

References

1. Hong, X.; Dalouchi, F.; Prom, J.C.; Ponte, M.E.; Hockey, B.L.; Braun, J.L.; Nies, P.; Stoltz, A.; Chakravarthi, V.P.; Sinha, M.; et al. Female reproductive dysfunction and transgenerational consequences following prolonged spaceflight exposure. *Proc. Natl. Acad. Sci. USA* **2026**, *123*, e2606092123. https://doi.org/10.1073/pnas.2606092123.
2. Benjamini, Y.; Hochberg, Y. Controlling the False Discovery Rate: A Practical and Powerful Approach to Multiple Testing. *J. R. Stat. Soc.* **1995**, *57*, 289–300. https://doi.org/10.1111/j.2517-6161.1995.tb02031.x.
3. Storey, J.D.; Tibshirani, R. Statistical significance for genomewide studies. *Proc. Natl. Acad. Sci. USA* **2003**, *100*, 9440–9445. https://doi.org/10.1073/pnas.1530509100.
4. Storey, J.D. A direct approach to false discovery rates. *J. R. Stat. Soc.* **2002**, *64*, 479–498. https://doi.org/10.1111/1467-9868.00346.
5. Quiros, P.M.; Goyal, A.; Jha, P.; Auwerx, J. Analysis of mtDNA/nDNA Ratio in Mice. *Curr. Protoc. Mouse Biol.* **2017**, *7*, 47–54. https://doi.org/10.1002/cpmo.21.
